# Realizing the potential of telemedicine in global health

**DOI:** 10.7189/jogh.09.020307

**Published:** 2019-12

**Authors:** Taehoon Kim, James E Zuckerman

**Affiliations:** Harvard Medical School, Brigham and Women’s Hospital, Boston, Massachusetts, USA

In December 2017, for the *first* time in history, private citizens of North Korea teleconferenced with foreigners via the internet as part of an online medical education program. This telemedicine initiative is connecting medical professionals from around the world with their North Korean counterparts to revamp the country’s challenged medical education systems [1]. While these developments appear to contradict the country’s longstanding geopolitical stances, especially those of censoring access to foreign educational resources as well as severely limiting communication with foreigners, they are the government’s practical response to the deleterious effects these policies have had on its health care systems. This telehealth initiative is just one of many examples illustrating the singular promise of telemedicine to improve the health of even the most remote communities around the world, showcasing its ability to defy physical, geopolitical, economic, and social distances.

Telemedicine, defined as the provision of health care remotely via information and communications technology (ICT), originated as early as in the 20^th^ century. Despite its early founding and potential, technological and financial barriers associated with transmitting video, audio, and images via nascent telecommunications infrastructure predictably limited its widespread implementation [2]. It is therefore not surprising that telemedicine was not initially envisioned as a tool for tackling global health challenges that plagued areas with poor infrastructure and lack of resources – the very factors that impeded widespread implementation in industrialized nations [3]. Indeed, one might assume that in resource-poor settings, lack of funding, technical resources, and infrastructure still hinder its deployment worldwide.

However, in the last few decades, there have been significant advancement of ICT, reduction in associated costs, and growing prevalence of technological resources even in resource-poor settings [3,4]. First, despite competing funding priorities, governmental and non-governmental funding has significantly increased in the last decade. In fact, disbursement of mobile health funding to developing countries has steadily grown, with over two hundred million dollars being pledged in 2010 alone [4]. Second, in a similar vein, infrastructures required for successful adoption of telemedicine initiatives have continually improved in developing nations. In fact, according to the World Health Organization, four in five developing nations throughout the world now offer at least one type of mobile health program to deliver essential health services to the population, which range from health call centers to emergency and disaster relief programs [3]. Third, regarding access to internet and mobile phone penetration, according to the International Telecommunications Union and the Global System for Mobile Communications Association, commercial wireless signals cover over 90% of the world’s population, extending far beyond the reach of the electrical grid. As an example, 89% of rural regions in South Africa, Mauritius, Kenya, and Malawi now have access to the Internet [3,4].

These exciting developments demand that medical communities around the world investigate the potential of telemedicine to confront profound global health issues as well as the challenges that still hinder its deployment worldwide. In this viewpoint article, we discuss the potential inherent in leveraging mobile health to deliver essential health care services in under-resourced settings.

Telemedicine can significantly improve health care delivery for patients with limited access to medical services. Indeed, telehealth programs can cost-effectively provide services, from radiology to dermatology, to at least some of the millions patients who lack adequate health care [3,5]. The expected scarcity of health professionals, especially specialists, in developing countries demands that low- and middle-income countries use novel approaches to improve access in the near future [6].

Moreover, the global trend in the rise of chronic diseases across income groups suggests that we need to do more than merely improve access. The problem demands patient engagement and education. Mobile health applications can facilitate treatment support, disease management, and patient self-care, educating populations about their conditions and effecting positive behavioral changes [4]. Furthermore, telehealth programs can avoid costly hospitalizations and can help patients reach health goals through remote patient monitoring and incorporating patients and family members into the care process [3]. As many of the leading global risk factors become increasingly behavioral in nature, the need to inform patients about their health behaviors and conditions rises.

Developing nations can also leverage telemedicine to improve weak public health systems that are under cost constraints. Such systemic challenges are often deep-rooted and multi-sectoral, stemming from weak supply chains, poor information systems, lackluster management of human resources, and unreliable financing mechanisms [3]. Telehealth initiatives could serve as both reliable and inexpensive substitutes for these sectors. While there may be concerns that telemedicine requires established infrastructure and technological resources to perform such system-wide functions, in many cases, this is only a perception. In fact, even with limited resources, telehealth applications can increase operational and organizational efficiencies of existing systems, helping to reduce health care costs and improve health outcomes [3,4]. Telemedicine offers a potential solution to rein in costs while effectively performing vital public health functions.

Telehealth applications cannot only improve health care systems but also create an interconnected global health network responsive to humanitarian crises. Inexpensive yet dependable programs can perform surveillance and monitoring of medical emergencies, generating health data to inform international aid programs and policies [7]. Telemedicine is a viable response to the need for an interconnected network of data sharing as well as funding for international crises.

**Figure:**
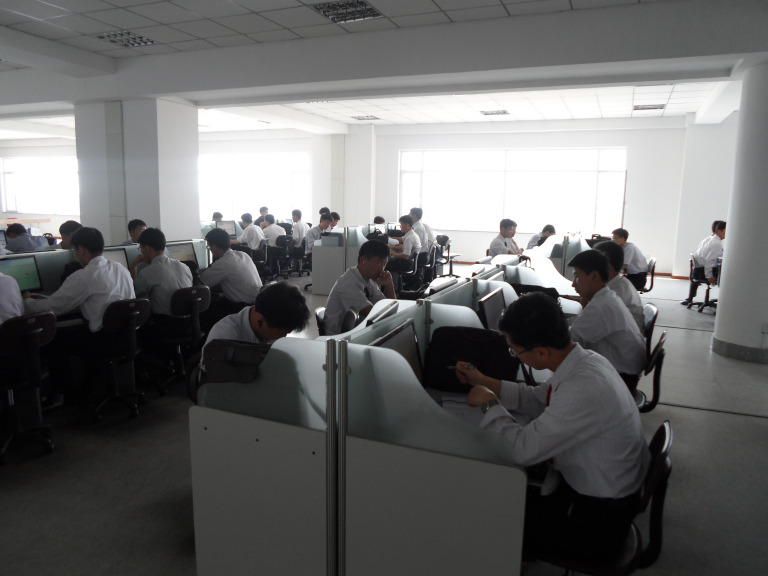
Photo: From the collection of DoDaum, used with permission.

Despite its promise, telemedicine still faces many barriers to wide-scale acceptance in resource-limited settings. The problems inhibiting telemedicine’s adoption are multifactorial, some of which include: lack of transnational regulatory standards, biases against technology-based medicine, absence of policy changes, and little to no interoperability between initiatives, and inertia [3,4]. To truly realize the promise of telehealth, therefore, a multipronged approach must be employed to confront what are inherently interdisciplinary challenges to its diffusion.

First, research communities must explore sustainable models of telehealth programs that can become integrated into existing health care systems. While increased emphasis on effectiveness and cost-effectiveness research would facilitate the uptake of telemedicine by informing policies and investment decisions, this should not be the only approach. Although supply-induced innovation of telehealth has led to diverse programs, there has not been critical investigation into how to logistically and fiscally sustain such initiatives in resource-poor settings. Certainly, such investigations ought to also consider educational and functional counterparts for telemedicine deployments. Without carefully researching these aspects that may buttress the implementation of telehealth programs, larger-scale and sustainable deployments may ultimately be limited. This new emphasis and the ensuing exploration may help to produce a cultural shift in how telemedicine is perceived, from one of mere novelty to one of integral function in developing nations.

Second, progressive leadership at the international, national, and local levels must establish policies that coalesce standards and regulations into coherent visions for telehealth. At the international level, specialized and multinational agencies should set broad and comprehensive regulatory and legal frameworks for telemedicine programs that cross borders. National or local governments must supplement such policies to accord with local health, legal, sociocultural, and financial needs. These measures will shift telehealth from its current fragmented system of innovation and implementation to one that benefits from uniform standards within which funders, governments, and private entities can deploy their telemedicine programs. Additionally, such environments with uniform guidelines will help researchers better conduct and publish rigorous scientific studies, thereby improving the evidence base that can further refine policies. In essence, top-down policy changes impelled by sensible leadership are necessary to establish standards upon which to implement, evaluate, and drive need-based innovations.

Third, strategic partnerships between diverse stakeholders are warranted to create telehealth applications that can truly benefit health care systems. Collaborative initiatives that include governments, private sectors, and international agencies may help to create programs that are more interoperable, allowing such platforms to become interdependent parts of health care sectors that can perform and integrate functions across different programs. These partnerships, however, may occur more often in predictable environments established by international and national leadership. The increase in collaborations may further motivate other nations and local governments to adopt sensible policies, thus spurring a virtuous cycle of adoption by others.

While telemedicine can and should serve critical roles in global health, it is not a panacea. Indeed, telehealth cannot and should not replace the sanctity of the patient-provider relationship – the basis of good medicine. However, it ought to fill in current gaps in resource-poor health care systems and tackle profound challenges to health. We are at a crossroads where supply-induced innovations in telemedicine must be guided by sensible and multifaceted approaches if telehealth applications are to be successful. If the chosen path is trod thoughtfully, we may be able to realize the promise of telehealth and truly improve health care access and quality for the world’s underserved populations. As an example, despite the number of political and diplomatic rows that have occurred since its start, the online medical education program in North Korea has thrived [8]. As of mid-2019, it has expanded nationwide, serving as a testament to what might be feasible throughout resource-poor regions worldwide in the setting of robust international partnerships, supportive domestic policies, continued research, and creativity.
